# Biopreservation of *Hericium erinaceus* By-Products via Lactic Acid Fermentation: Effects on Functional and Technological Properties

**DOI:** 10.3390/foods14213721

**Published:** 2025-10-30

**Authors:** Mafalda Silva, Manuela Vida, Ana Cristina Ramos, Luísa Cristina Roseiro, Nuno Alvarenga, Sandra Gomes, Fernando C. Lidon, Fernando H. Reboredo, Elsa M. Gonçalves

**Affiliations:** 1 UTI—Unidade de Tecnologia e Inovação, Instituto Nacional de Investigação Veterinária e Agrária, Av. da República, Quinta do Marquês, 2780-157 Oeiras, Portugal; mafaldasdos@gmail.com (M.S.); manuela.vida@iniav.pt (M.V.); cristina.ramos@iniav.pt (A.C.R.); cristina.roseiro@iniav.pt (L.C.R.); nuno.alvarenga@iniav.pt (N.A.); sandra.gomes@iniav.pt (S.G.); 2 Faculdade de Ciências e Tecnologia, Universidade Nova de Lisboa, Campus da Caparica, 2829-516 Caparica, Portugal; fjl@fct.unl.pt (F.C.L.); fhr@fct.unl.pt (F.H.R.); 3 GeoBiotec—GeoBioTec Research Institute, Faculdade de Ciências e Tecnologia, Universidade Nova de Lisboa, Campus da Caparica, 2829-516 Caparica, Portugal

**Keywords:** lactic acid bacteria, *Hericium erinaceus*, by-products, biopreservation, bioactive content

## Abstract

Lactic acid fermentation is an effective strategy for food preservation and functional enhancement. This study evaluated the fermentation of *Hericium erinaceus* by-products using *Lactoplantibacillus plantarum* and *Lactocaseibacillus rhamnosus*, assessing microbial stability, physicochemical parameters, phenolic content, antioxidant activity, rheology, and biogenic amine formation over 240 h. *Lp. plantarum* promoted rapid acidification, reducing the pH from 6.0 to 4.65 within 72 h, while *Ls. rhamnosus* reached its lowest value of 3.8 at 144 h. Both strains effectively inhibited spoilage organisms:* Lp. plantarum* suppressed yeasts, molds, and *Pseudomonas* spp. by 144 h, whereas the control reached >6.0 log CFU/g of Pseudomonas at 240 h. Fermentation altered the texture, with the storage modulus (G′) decreasing from ~17 kPa to <3 kPa. Functional enrichment was also observed, with total phenolic content increasing from 32 to 48 mg GAE/100 g and antioxidant activity (DPPH) reaching 2562 µmol TE/100 g compared with 1954 µmol TE/100 g in the control. Importantly, cadaverine accumulated to 70.3 mg/kg in the control but remained below 15 mg/kg in inoculated samples, while spermidine was consistently higher in *Lp. plantarum*-treated mushrooms (~45 mg/kg). These results demonstrate that lactic acid fermentation can transform perishable *H. erinaceus* by-products into safe, stable, and bioactive ingredients, supporting their application in functional foods, nutraceuticals, and clean-label products while contributing to circular bioeconomy goals.

## 1. Introduction

Biopreservation refers to the use of beneficial microorganisms and their metabolites to enhance the safety, shelf life, and quality of foods [1]. This approach is increasingly valued as consumers demand natural alternatives to chemical preservatives and as the food sector embraces sustainable, clean-label technologies. Among these strategies, fermentation has long been recognized as a natural and effective method for the preservation and biotransformation of diverse substrates, ranging from milk and cereals to vegetables, fruits, and meat products [2,3,4,5,6].

Lactic acid bacteria (LAB) are central to these processes. By converting carbohydrates into organic acids, primarily lactic acid, and producing antimicrobial metabolites such as bacteriocins and exopolysaccharides, LAB inhibit spoilage and pathogenic microorganisms, thereby extending shelf life [7,8]. Many LAB strains also hold “Generally Recognized As Safe” (GRAS) or Qualified Presumption of Safety (QPS) status [9,10]. Importantly, fermentation does not merely preserve food—it can also enhance nutritional and functional properties, contributing to the development of foods with antioxidant, anti-inflammatory, or neuroprotective potential [11].

In recent years, there has been growing scientific and industrial interest in applying LAB fermentation to valorize agri-food by-products, which represent a major component of global food waste. Fermenting plant, fruit, and mushroom residues not only prevents environmental burden but also generates bioactive ingredients with added nutritional and functional value. For example, Bartkiene et al. [5] demonstrated that lactic fermentation of *Boletus edulis* and *Cantharellus cibarius* improved microbial safety and antioxidant capacity, while Kalinowska et al. [12] and Leonard et al. [13] reported similar findings for apple pomace and plant-based matrices, respectively. Moreover, Cheng et al. [14] showed that fermenting blueberry pomace with LAB enhanced phenolic release and short-chain fatty acid production, and Sarıtaş et al. [15] highlighted how fermentation broadly increases antioxidant potential across diverse food substrates. These examples demonstrate the versatility of LAB fermentation as a sustainable valorization pathway within the circular bioeconomy.

*Hericium erinaceus* (lion’s mane mushroom) is a medicinal fungus distinguished by its content of hericenones and erinacines, compounds associated with antioxidant, anti-inflammatory, and neuroprotective effects [16]. However, due to its high moisture content, *H. erinaceus* is highly perishable and difficult to store or distribute [17]. Lactic acid fermentation of common edible mushrooms such as *Agaricus bisporus* and *Lentinula edodes* has been investigated [5,18]. Considering that mushroom fruiting bodies are rich in carbohydrates (32–61% of dry matter) [19], these residues represent highly promising substrates for microbial fermentation aimed at improving stability and unlocking additional functional properties.

In addition to microbial stability and functional enrichment, fermentation also affects several quality parameters of the substrate, including acidity, color, texture, and phenolic composition [20,21,22,23]. These changes not only influence preservation but also define the technological and sensory characteristics of the final product. Another critical aspect is the formation of biogenic amines (BAs), nitrogenous compounds generated through microbial decarboxylation of amino acids [24,25], While some BAs, such as histamine and tyramine, are associated with toxicological risks, others like spermidine and spermine may exert beneficial physiological functions, including antioxidant and autophagy-inducing activities [26,27,28]. Thus, evaluating BA dynamics during fermentation, alongside physicochemical and functional traits, provides a more comprehensive assessment of the safety and overall quality of fermented foods.

In this context, the present study contributes to the current efforts toward sustainable food systems by investigating lactic acid fermentation as a biopreservation and valorization strategy for *H. erinaceus* by-products. By applying *Lactobacillus plantarum* and *Lactobacillus casei*, we examine their effects on microbial stability, physicochemical parameters, phenolic transformation, antioxidant capacity, and rheological properties. This work not only introduces a novel approach for mushroom waste valorization but also aligns with circular bioeconomy principles by converting perishable residues into safe, stable, and bioactive ingredients for functional food and nutraceutical applications.

## 2. Materials and Methods

### 2.1. Mushroom Pre-Treatment and Preparation

Fresh fruiting bodies of *Hericium erinaceus* were obtained in March 2025 from “Cogumelos Serra da Lua” (Porto de Mós, Portugal; GPS coordinates: 39.47870, −8.85636), an indoor mushroom cultivation facility. The samples used in this study were considered byproducts of the production process, consisting of mushrooms that were physically damaged or otherwise unsuitable for commercial sale. As such, they represent a potential source of food waste.

Upon arrival at the laboratory, the mushrooms were carefully cleaned using paper towels to remove residual dirt. Prior to fermentation, the samples underwent a standardized thermal pre-treatment intended to inactivate endogenous enzymatic activity and reduce the indigenous microbial population. Specifically, the mushrooms were sealed in heat-resistant, food-grade plastic bags and subjected to blanching in boiling water for 3 min, a condition optimized in preliminary trials to ensure enzymatic inactivation.

Subsequently, blanched mushrooms were homogenized using a high-speed homogenizer (ULTRA-TURRAX^®^ T25 basic, IKA^®^-WERKE, Staufen, Germany) at 16,000 rpm for 1 min with 2% (*w*/*w*) sodium chloride (NaCl) to enhance microbial selectivity. The resulting homogenate was then aseptically divided into three equal portions of 500 ml each, Control (C), *Lactocaseibacillus rhamnosus* (*Lc*), and *Lactoplantibacillus plantarum* (*Lp*), for subsequent inoculation procedures (Section 2.2).

### 2.2. Strains Preparation, Inoculation and Microbiology Analysis

The lactic acid bacteria (LAB) strains employed in this study—*Lactoplantibacillus plantarum* C1170 and *Lactocaseibacillus rhamonosus* C243—were obtained from the bacterial strain collections of the Instituto Nacional de Investigação Agrária e Veterinária (INIAV), Portugal. Each strain was cultivated in de Man, Rogosa and Sharpe (MRS) broth at 30 °C under aerobic conditions. Subsequently, the culture is transferred to MRS agar medium and incubated at 30 °C for 72 h. After this step, the bacterial suspension is adjusted to a concentration of 1.5 × 10^9^ CFU/mL, corresponding to 5 on the McFarland scale.

The nomenclature of the lactic acid bacteria used in this study follows current international recommendations for microbial taxonomy. The strains are designated *Lactiplantibacillus plantarum* and *Lacticaseibacillus rhamnosus*. For convenience, and after their first full mention, these species are abbreviated as *Lactiplantibacillus plantarum* (*Lp. plantarum*) and *Lacticaseibacillus rhamnosus* (*Ls. rhamnosus*). In figures and tables, the compact two-letter codes (*Lp* and *Lc*) are used to ensure consistency with the graphical labels and to maintain clarity throughout the text.

Following the pre-treatment described in Section 2.1, the mushroom homogenate was aseptically divided under a laminar flow hood (NU-201 4 ft, NuAire, Plymouth, MN, USA), into three equal portions of 500 mL each. Two portions were inoculated with 33.3 mL of either *Lp. plantarum* C1170 or *Ls. rhamnosus* C243 to achieve an initial bacterial concentration of approximately 10^8^ CFU/g of sample. The third portion was left uninoculated to serve as a control (spontaneous fermentation). Aliquots were prepared in triplicate for each variable and time point. All fermentations were conducted under aerobic conditions at 25 ± 1 °C for 240 h, with agitation at 100 rpm. To monitor microbial dynamics, 10 g samples were aseptically collected from each replicate at five time points: 0 h, 24 h, 72 h, 144 h, and 240 h. Sampling was performed using a flame-sterilized knife and clamp to ensure asepsis. Microbiological analyses included the enumeration of total viable mesophilic microorganisms at 30 °C, performed in accordance with ISO 4833-1 [29]. Lactic acid bacteria (LAB) were quantified following ISO 15214:1998 [30], which specifies colony-count techniques using selective media under aerobic conditions. *Pseudomonas* spp. were enumerated using ISO 13720:2010 [31]. Yeasts and molds were determined according to ISO 21527-1 [32]. Microbial counts were expressed as logarithmic values of colony-forming units per gram of sample (log CFU/g).

### 2.3. Physicochemical and Functional Analyses

#### 2.3.1. pH, TA, TSS Determination

Titratable acidity (TA), expressed as grams of lactic acid (LA) per 100 g of sample, was determined following the NP-1421 standard using an automatic titrator (665 Dosimat, Metrohm, Herisau, Switzerland). For this analysis, 5 g of mushroom from each treatment group was homogenized in ultrapure water to a final volume of 50 mL using a high-speed homogenizer (ULTRA-TURRAX^®^ T25 basic, IKA^®^-WERKE) at 16,000 rpm for 1 min. The pH of the homogenate was measured prior to titration using a calibrated digital pH meter (Crison Micro pH 2001, Crison Instruments, Barcelona, Spain) standardized with pH 4.0 and 7.0 buffers.

Total soluble solids (TSS) were assessed using a digital refractometer (Atago DDR-A1, ATAGO Co., Ltd., Tokyo, Japan), and results were expressed as a percentage (%).

#### 2.3.2. Color Characterization

The color characteristics of the fermented product were determined using a Minolta CR-300 colorimeter (Osaka, Japan) following the CIE Lab* color space. In this system, the L* coordinate represents lightness, ranging from 0 (black) to 100 (white); the a* coordinate expresses the green–red axis (negative values for green and positive for red); and the b* coordinate describes the blue–yellow axis (negative values for blue and positive for yellow) [19]. The instrument was calibrated using a white tile standard. Color measurements were taken on the surface of each triplicate at four different locations for each box, a total of 8 determinations for each day. The total color difference (∆E) between the pre-fermentation and post-fermentation was calculated following:

(1) $$∆E=\sqrt{{\left(L^{*}-L_{0}^{*}\right)}^{2}+{\left(a^{*}-a_{0}^{*}\right)}^{2}+{\left(b^{*}-b_{0}^{*}\right)}^{2}}$$

#### 2.3.3. Rheology Measurements

Small-amplitude oscillatory shear (SAOS) tests were performed using a controlled-stress rheometer (Haake Mars iQ Rotational Rheometer, Thermo Fisher Scientific, Karlsruhe, Germany) equipped with an air-cooled Peltier temperature control module, following a previously described protocol with minor adaptations to the matrix [33]. Measurements were conducted at 20 ± 1 °C using a serrated parallel plate geometry (20 mm diameter) with a fixed gap of 1.5 mm. Excess sample was carefully trimmed with a plastic blade, and the loaded sample was equilibrated for 5 min prior to testing. To define the linear viscoelastic region (LVR), a preliminary stress sweep (0.6–1000 Pa) was performed at a constant oscillation frequency of 1 Hz on separate aliquots of each sample. Based on the identified LVR, dynamic frequency sweep tests were then conducted at a constant stress (3, 10, or 100 Pa, depending on the sample), within the established LVR, over the frequency range of 0.01–100 Hz. The main rheological parameters recorded were the storage modulus (G′, Pa) and the loss modulus (G″, Pa), which were plotted as a function of frequency to generate the mechanical spectra. Quantitative comparisons among samples were based on the storage modulus values at 1 Hz. All measurements were carried out in triplicate, and the applied shear stress for each test was selected according to the sample-specific LVR.

#### 2.3.4. Determination of Total Phenolic Content and Antioxidant Capacity

Methanolic extracts were obtained by combining 2.5 g of mushroom tissue with 10 milliliters of methanol for each sample. The total phenolic content (TPC) was assessed using a modified Folin–Ciocalteu protocol [34]. Specifically, 150 μL of each extract was diluted with 2400 μL of nanopure water in test tubes, followed by the addition of 150 μL of 0.25 N Folin–Ciocalteu reagent. After a 3 min incubation, 300 μL of 1 N sodium carbonate (Na_2_CO_3_) was added. The mixtures were then kept in the dark at room temperature for 2 h. Absorbance was measured at 725 nm using a JASCO V-530 UV/VIS spectrophotometer (Jasco International, Tokyo, Japan). TPC values were reported as milligrams of gallic acid equivalents (GAEs) per 100 g of fresh weight (fw).

To evaluate antioxidant activity, three assays were conducted: DPPH, ABTS, and FRAP, following modified procedures from Yu et al. [35] and Pereira et al. [36].

For the DPPH assay, a stock solution was prepared by dissolving 24 mg of DPPH in 100 mL of methanol and then diluted to reach an absorbance of 1.1 ± 0.02 at 515 nm. In each cuvette, 2850 μL of the DPPH solution was mixed with 150 μL of either a Trolox standard, sample, or methanol (as a blank). Absorbance at 515 nm was recorded after a 2 h incubation, and antioxidant capacity was expressed as micromoles of Trolox equivalents per 100 g of fresh weight (μmol TE/100 g fw).

The ABTS assay involved mixing equal volumes of 4.8 mM potassium persulfate and 14 mM ABTS, allowing the mixture to react in the dark at room temperature for 16 h. The resulting solution was diluted to an absorbance of 1.1 ± 0.02 at 734 nm. For each measurement, 2850 μL of the ABTS solution and 150 μL of sample or standard were combined in cuvettes, incubated for 2 h in the dark, and absorbance was measured at 734 nm. Results were reported as μmol TE/100 g fw.

For the FRAP assay, the reagent was prepared by mixing acetate buffer (0.3 M, pH 3.6), TPTZ (10 mM in 40 mM HCl), and 20 mM ferric chloride in a 10:1:1 ratio. In each cuvette, 0.2 mL of sample, FeSO_4_·7H_2_O standard, or distilled water (blank) was added, followed by 1.8 mL of the FRAP reagent. Samples were incubated in the dark for 30 min, after which absorbance was measured at 593 nm. The antioxidant capacity was expressed as millimoles of FeSO_4_·7H_2_O per 100 g of fresh weight.

#### 2.3.5. Determination of Biogenic Amines

Biogenic amines (BAs) were quantified by high-performance liquid chromatography (HPLC) according to Roseiro et al. [37], with modifications. Briefly, 5 g of sample was homogenized in 25 mL of 0.4 M perchloric acid using a Polytron PT 3100 homogenizer (Kinematica AG, Luzern, Switzerland) at 5000 rpm for 1 min. The homogenate was centrifuged at 10,000 rpm for 10 min at 4 °C, and the supernatant was filtered through Whatman filter paper. The extract was adjusted to a final volume of 50 mL with 0.4 M perchloric acid.

For derivatization, 1 mL of the extract was mixed with 1 mL of dansyl chloride solution (5 mg/mL in acetone) and incubated at 40 °C for 40 min in the dark. The reaction was stopped by cooling on ice, and the mixture was adjusted to 5 mL with acetonitrile. Samples were filtered through Acrodisc GHP 0.45 µm membranes (Gelman Sciences, Ann Arbor, MI, USA) before injection.

HPLC analysis was performed using a Waters Alliance 2695 separation module (Waters Corporation, Milford, MA, USA) coupled to a Waters 2996 diode array detector (DAD) (Waters Corporation, Milford, MA, USA). Separation was achieved on a Spherisorb ODS2 C18 column (250 × 4.0 mm, 5 µm particle size) maintained at 30 °C. The mobile phase consisted of solvent A (0.1 M ammonium acetate) and solvent B (acetonitrile), using a linear gradient from 50% to 90% B over 19 min, followed by a 10 min equilibration. The flow rate was set at 1.0 mL/min, injection volume was 20 µL, and detection was carried out at 254 nm.

The following amines were quantified: tryptamine, β-phenylethylamine, putrescine, cadaverine, histamine, tyramine, spermidine, and spermine. Quantification was based on external calibration curves prepared with analytical standards (Sigma-Aldrich, St. Louis, MO, USA) across a concentration range of 0.5–20 mg/L, with correlation coefficients (R^2^) > 0.99. Results were expressed as mg/kg of fresh weight (fw).

### 2.4. Statistical Analysis

All experiments were performed in triplicate. Statistical analyses were conducted using one-way analysis of variance (ANOVA) with Statistica™ software, version 8.0 [38]. Tukey’s Honest Significant Difference (HSD) test was applied at a significance level of *p* < 0.05 to assess differences among the mean values of mushroom properties across all time points. Results are presented as mean values ± standard deviation (SD).

## 3. Results

### 3.1. Dynamic of Microbiological Loads

**Figure 1:**
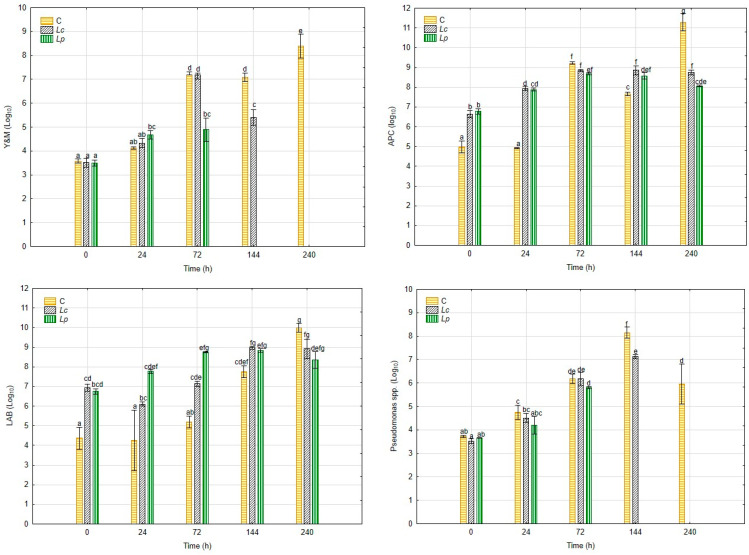
Microbiological analysis of *He* by-products, at different sampling times, 0 h, 24 h, 72 h, 144 h and 240 h. M Results are expressed as mean values ± standard deviation from triplicate measurements (*n* = 3). Within each column, mean values followed by different letters indicate statistically significant differences (*p* < 0.05).

The microbiological profile of *H. erinaceus* by-products during fermentation is shown in Figure 1. At the beginning of the process (0 h), aerobic plate counts (APC) were similar across all treatments, averaging around 4.5 log CFU/g. In the inoculated samples, both *Lp. plantarum* (*Lp*) and *Ls. rhamnosus* (*Lc*) triggered a rapid increase in microbial load, reaching approximately 7.0 log CFU/g after 24 h, while the control sample showed only a moderate increase. By the end of fermentation (240 h), APC values stabilized at 7.5–7.7 log CFU/g in the inoculated groups, whereas the control remained lower, around 6.5 log CFU/g.

Lactic acid bacteria (LAB) counts reflected the inoculation strategy. At time zero, *Lp* and *Lc* treatments showed initial values of 6.8 log CFU/g, compared to 4.4 log CFU/g in the control. In inoculated groups, LAB populations increased slightly until 72 h, then gradually declined towards 240 h, although remaining dominant within the microbiota. In the control, however, LAB increased steadily over time, reaching ~6.0 log CFU/g by the end of the fermentation.

Yeasts and molds (Y&M) presented initial counts close to 3.5 log CFU/g in all treatments. In the control, levels rose continuously, exceeding 7.0 log CFU/g after 72 h and remaining high until 240 h. A similar increase was observed in the *Lc* treatment up to 144 h, but counts dropped sharply thereafter, reaching ~2.0 log CFU/g at 240 h. In contrast, *Lp* completely suppressed Y&M by 144 h, with no detectable colonies at later time points.

The evolution of *Pseudomonas* spp. followed a comparable pattern. Starting from ~3.6 log CFU/g, counts in the control increased steadily to more than 6.0 log CFU/g at 240 h. In the *Lc* treatment, Pseudomonas grew until 144 h, after which a strong reduction was observed, with final values close to the detection limit. The *Lp* treatment showed earlier and more efficient suppression, with counts dropping markedly after 72 h and becoming undetectable at 144 h and 240 h.

### 3.2. Physicochemical and Functional Properties

#### 3.2.1. pH, TA, and TSS of the Fermented Products

**Figure 2:**
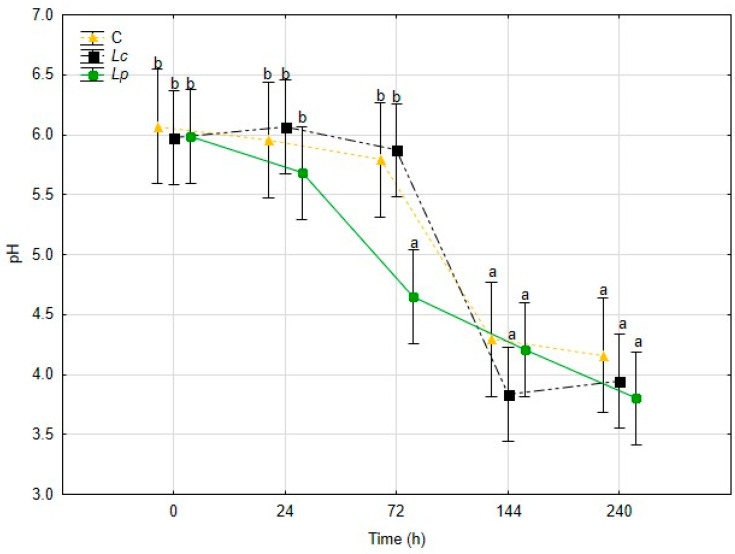
pH variation during 240 h of fermentation, for the three different treatments. Means values ± standard deviation of triplicate (n = 3). Different letters are statistically different (*p* < 0.05).

The evolution of pH during fermentation is presented in Figure 2. The initial value of the mushroom preparation was 6.00 ± 0.05, with no differences among treatments. In the samples inoculated with *Lp. plantarum* (*Lp*), a rapid decline was observed, with pH reaching 4.65 after 72 h. A further reduction occurred in the later stages, stabilizing below 4.5 at 240 h. The *Lc* treatment also showed a marked pH decrease, reaching 4.80 at 72 h and 3.80 at 144 h, the lowest value among all treatments. In contrast, the control sample displayed a much slower decrease, with pH values remaining above 5.0 until 144 h and only approaching 4.6 at the end of fermentation. These results confirm that inoculated LAB strains accelerated acidification compared to spontaneous fermentation, with *Lp* showing a faster early response, while *Lc* produced a more pronounced acidification in the later stages.

Table 1 summarizes the changes in total soluble solids (TSS) and titratable acidity (TA). At the beginning of the experiment, TSS values were similar among treatments, ranging from 7.4% in the control to 7.8% in the Lc sample. During fermentation, a slight increase was observed in inoculated groups, peaking at 8.8% in *Lp* and 8.6% in *Lc* at 144 h, compared to 7.8% in the control. By the end of the process, all treatments converged around 8.4%, suggesting that sugars initially solubilized during early fermentation were progressively consumed, leading to stabilization.

Titratable acidity showed more distinctive differences between treatments. At 0 h, all groups presented similarly low values (~0.2 g LA/100 g). After 72 h, inoculated samples displayed a significant increase, particularly in *Lp*, which reached 0.7 g LA/100 g, compared to 0.3 g LA/100 g in both the control and *Lc*. From this point onward, acidification progressed steadily in all treatments. At 240 h, the highest TA values were recorded in inoculated samples, with 1.7 g LA/100 g in *Lc* and 1.6 g LA/100 g in *Lp*, while the control reached only 1.4 g LA/100 g.

Together, these results demonstrate that inoculation with LAB enhanced acid production and accelerated the decline in pH compared to spontaneous fermentation. *Lp* ensured faster early acidification, which is particularly relevant for microbial safety, whereas *Lc* drove stronger late-stage acid accumulation. The combined analysis of pH, TSS, and TA highlights the efficiency of both inoculated strains in metabolizing mushroom carbohydrates into organic acids, thereby stabilizing the substrate and improving its preservation potential.

#### 3.2.2. Color Evaluation

The color parameters (L*, a*, b*) and total color difference (∆E) of *H. erinaceus* by-products during fermentation are presented in Table 2. At the initial time (0 h), all treatments showed relatively high lightness values (L*), ranging from 67.7 in the control to 72.5 in the *Lp* treatment, indicating a pale, light-colored matrix. The redness parameter (a*) was close to zero (1.2–1.5), and yellowness (b*) varied between 27.8 and 29.8, reflecting the natural whitish-beige color of the mushroom.

After 24 h, inoculated samples exhibited slightly higher L* values (73.3 in *Lp* and 72.4 in *Lc*) compared to the control (71.8), accompanied by an increase in redness and yellowness. The most pronounced changes occurred at 72 h, when both inoculated treatments presented significant increases in a* and b* values. For instance, *Lc* reached a* = 4.3 and b* = 35.2, compared to a* = 3.0 and b* = 33.2 in the control. These changes suggest that microbial metabolism during early fermentation intensified color saturation.

From 144 h onward, however, lightness decreased gradually in all treatments, indicating a darkening effect. By 240 h, L* values converged across treatments, with the control at 61.7, *Lp* at 61.6, and *Lc* at 60.8. At the same time, redness and yellowness values dropped back toward their initial levels, with a* ranging from 1.1 to 1.7 and b* from 26.7 to 27.7.

The total color difference (∆E) reflected these changes over time. At 24 h and 72 h, the ∆E values were moderate, ranging from 4.1 to 8.3 depending on the treatment. At 144 h, the control displayed only a minor difference (1.7), while both inoculated samples maintained intermediate values (~5). By the end of fermentation, however, inoculated groups exhibited the highest ∆E values (11.4 for *Lp* and 11.0 for *Lc*), almost double the control (6.4).

These results indicate that LAB fermentation induced more pronounced color modifications than spontaneous fermentation. Early increases in redness and yellowness were followed by a gradual darkening of the samples, leading to significant overall differences at 240 h. While these changes are likely linked to acidification and phenolic oxidation, they may also affect consumer perception, since a darker appearance is often associated with quality loss in fresh mushrooms.

#### 3.2.3. Rheology Properties

**Figure 3:**
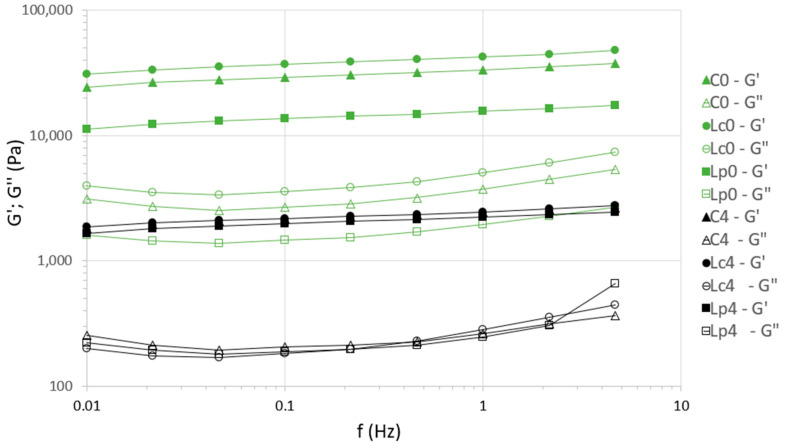
Variations in the storage modulus G′ (solid symbols) and loss modulus G″ (open symbols) in the frequency sweep curves of *Hericium erinaceus* samples fermented with LAB strains *Lc* and *Lp* compared to a non-inoculated control, measured at 0 (▲/△—C0, ●/○—*Lc*0, ■/□—*L*p0) and 244 h, (▲/△—C4, ●/○—*Lc*4, ■/□—*Lp*4).

The viscoelastic behavior of *H. erinaceus* by-products was evaluated by monitoring the storage modulus (G′) and loss modulus (G″) during frequency sweep tests (Figure 3). At the beginning of fermentation (0 h), all treatments exhibited high G′ values at 1 Hz, ranging from approximately 16.6 to 19.0 kPa, while G″ values were much lower. This indicates that the samples behaved predominantly as elastic gels, with a well-structured network capable of maintaining firmness.

After 240 h of fermentation, a drastic reduction in viscoelastic parameters was observed in all treatments. Storage moduli decreased to values between 1.8 and 2.8 kPa, while loss moduli increased slightly. This shift reflects a loss of structural rigidity and a transition toward more liquid-like behavior. The changes coincided with strong acidification (Section 3.2.1), indicating that the low pH and accumulation of organic acids destabilized the polysaccharides [1]. This approach is increasingly valued as consumers demand natural alternatives to chemical preservatives and as the food sector embraces sustainable, clean-label technologies. Among these strategies, fermentation has long been recognized as a natural and effective method for the preservation and biotransformation of diverse substrates, ranging from milk and cereals to vegetables, fruits, and meat products [2,3,4,5,6].

Lactic acid bacteria (LAB) are central to these processes. By converting carbohydrates into organic acids and primarily lactic acid, and producing antimicrobial metabolites such as bacteriocins and exopolysaccharides, LAB inhibit spoilage and pathogenic microorganisms, thereby extending shelf life [7,8]. Many LAB strains also hold “Generally Recognized As Safe” (GRAS) or Qualified Presumption of Safety (QPS) status [9,10]. Importantly, fermentation does not merely preserve food—it can also enhance nutritional and functional properties.

In summary, fermentation caused a marked reduction in viscoelasticity and gel strength, leading to a more liquid product. While this loss of firmness may limit applications requiring solid texture, it may be advantageous for developing liquid or semi-liquid foods such as soups, sauces, or fermented beverages, where a smoother consistency is desirable.

#### 3.2.4. Determination of Total Phenolic Content and Antioxidant Capacity

**Figure 4:**
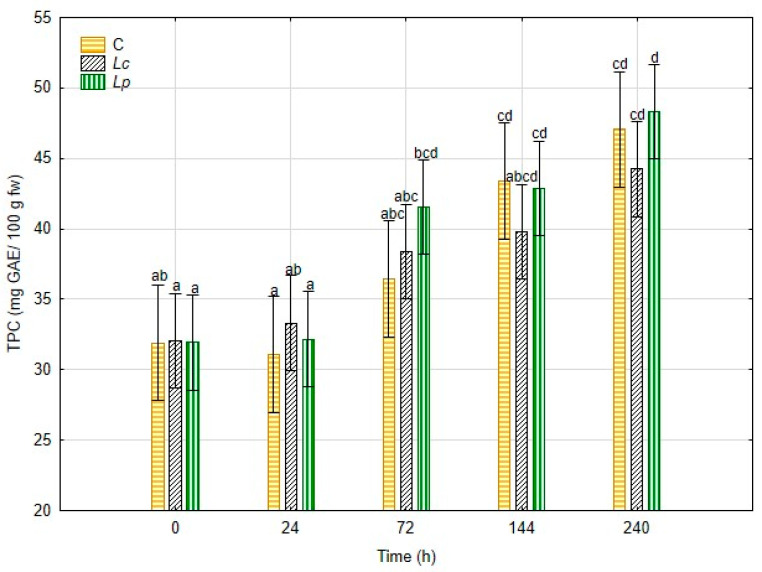
Total phenolic content (TPC) of *Hericium erinaceus* samples for 240 h fermentation. Different letters indicate statistically significant differences between treatments and time points (*p* < 0.05).

The total phenolic content (TPC) of *H. erinaceus* by-products during fermentation is shown in Figure 4.

At the beginning of the process (0 h), no significant differences were observed among treatments, with values close to 32 mg GAE/100 g fw. By 72 h, increases were detected across all groups, but were more pronounced in the inoculated samples: *Lp* reached ~41.5 mg GAE/100 g fw and *Lc* ~36.5 mg GAE/100 g fw, compared to ~34 mg GAE/100 g fw in the control. This trend continued throughout the process. At 144 h, the *Lp* treatment presented the highest TPC (42.9 mg GAE/100 g fw), followed by *Lc* (39.7 mg GAE/100 g fw) and the control (37.8 mg GAE/100 g fw). By the end of fermentation (240 h), phenolic content peaked at 48.3 mg GAE/100 g fw in *Lp* and 44.2 mg GAE/100 g fw in *Lc*, while the control reached 40.2 mg GAE/100 g fw. These results demonstrate that fermentation enhanced phenolic content, with *Lp* showing the strongest effect.

**Figure 5:**
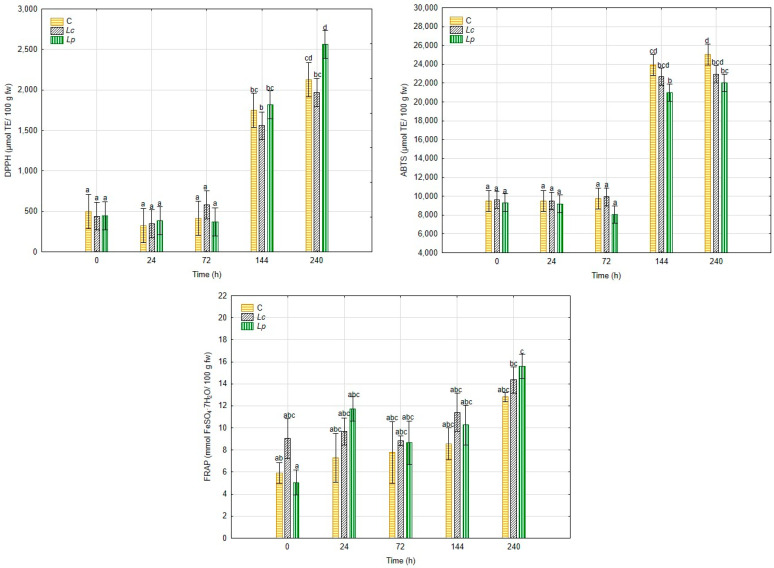
Antioxidant activity of *Hericium erinaceus* samples measured by DPPH, ABTS, and FRAP assays during 240 h of fermentation. Data are presented as mean ± standard deviation. Distinct letters denote statistically significant differences among the means (*p* < 0.05).

The antioxidant capacity, assessed by DPPH, ABTS, and FRAP assays, followed similar but assay-specific patterns (Figure 5). In the first 72 h, all treatments maintained relatively stable antioxidant values, indicating limited early changes. However, significant increases were recorded after 144 h. In the DPPH assay, radical scavenging activity rose markedly in inoculated treatments, with *Lp* reaching 2562 µmol TE/100 g fw and *Lc* 2318 µmol TE/100 g fw at 240 h, compared to 1954 µmol TE/100 g fw in the control.

The ABTS assay showed an even stronger response. At the end of fermentation, antioxidant activity in *Lc* and *Lp* treatments reached 22,972 and 22,030 µmol TE/100 g fw, respectively, while the control displayed a lower value of 19,482 µmol TE/100 g fw. This suggests that LAB metabolism enhanced the release of compounds with high ABTS radical scavenging capacity. A strong correlation (r = 0.94, *p* < 0.05) between ABTS and DPPH results supports the consistency of these findings.

In contrast, FRAP values showed a slower response. From 0 to 144 h, reducing power remained relatively stable across treatments, without significant differences. Only at 240 h were higher values detected in inoculated groups, with *Lp* showing the strongest effect (17.0 mmol FeSO_4_·7 H_2_O/100 g fw), followed by *Lc* (15.6 mmol FeSO_4_·7 H_2_O/100 g fw) and the control (13.9 mmol FeSO_4_·7 H_2_O/100 g fw).

Overall, fermentation led to a significant increase in TPC and antioxidant activity, particularly in inoculated treatments. The superior performance of *Lp* suggests a more efficient release or transformation of phenolic compounds, likely linked to enzymatic activity such as β-glucosidase, which can hydrolyze glycosidic bonds and liberate bound phenolics. The enhancement of antioxidant activity across complementary assays indicates that LAB fermentation contributes not only to microbial stability but also to functional enrichment of *H. erinaceus* by-products.

#### 3.2.5. Determination of Biogenic Amines

Table 3 presents the biogenic amine (BA) content of *H. erinaceus* by-products at the beginning and after 240 h of fermentation. In the control, BA accumulation was evident, particularly cadaverine, which increased from 15.1 to 70.3 mg/kg. Tryptamine levels also rose significantly (13.6 to 29.7 mg/kg), while phenylethylamine and histamine showed only minor variations. Tyramine decreased during fermentation but remained at relatively high levels compared to other amines.

In the samples inoculated with *Ls. rhamnosus*, BA concentrations remained generally stable over time. Cadaverine values did not exceed 10.5 mg/kg, and histamine and tyramine levels showed no marked changes from their initial values. The *Lp. plantarum* treatment also prevented major increases in BA content. Cadaverine remained low (13.0 mg/kg at 240 h), and histamine and tyramine showed only minor fluctuations. Notably, spermidine concentrations were higher in these samples at both initial and final time points, while spermine remained stable across treatments.

Overall, inoculated fermentations limited the accumulation of potentially harmful BAs compared with the control, while maintaining stable levels of naturally occurring polyamines.

## 4. Discussion

The results of this study demonstrate that controlled lactic acid fermentation is an effective strategy to stabilize and enhance *Hericium erinaceus* by-products, confirming both microbiological safety and functional enrichment.

Microbial analyses revealed that inoculation with LAB accelerated colonization and provided stronger control of spoilage organisms compared to spontaneous fermentation. *Lp. plantarum* (*Lp*) was particularly efficient, completely suppressing yeasts, molds, and *Pseudomonas* spp. by 144 h, whereas *Ls. rhamnosus* (*Lc*) required 240 h to achieve similar inhibition. Although Lc reached lower pH values, its delayed effect suggests that inhibitory activity is not exclusively linked to acidification. Previous studies have shown that *Lp* can produce antimicrobial metabolites such as bacteriocins and exopolysaccharides (EPS), which may act synergistically with organic acids to suppress spoilage microorganisms [39,40]. In contrast, indigenous microbiota in the control group failed to prevent microbial proliferation, reinforcing the importance of targeted inoculation [41,42].

Physicochemical changes confirmed active microbial metabolism. The decline in pH and concurrent increase in titratable acidity (TA) reflected organic acid accumulation, a well-established mechanism for pathogen inhibition in fermented foods [20,21,43,44]. Inoculated samples, particularly *Lp*, acidified more rapidly and extensively than the control, presenting a significant decrease (*p* < 0.05) after 72 h of fermentation reaching a final pH of 4.65, highlighting the superior metabolic performance of selected strains. The dynamics of total soluble solids (TSS), which rose slightly and then stabilized, indicate that soluble carbohydrates were first released and later consumed for acid production. Although this trend was more pronounced in inoculated samples, a gradual decrease in pH was also observed in the control, reflecting the contribution of native microbial metabolism, consistent with other fermentation systems [45].

Color parameters further illustrated the impact of fermentation. According to He et al. [22] the carbohydrate metabolism by LAB and consequent organic acid production and environmental acidification induces color alterations. All treatments exhibited relatively high L* values at the beginning of the fermentation process, indicating a light color. The progressive decrease in lightness (L*) and high total color difference (∆E) in inoculated groups suggest that LAB metabolism and acidification promoted oxidative browning reactions, possibly involving phenolic substrates. Similar observations have been reported in other mushroom fermentations [23]. By 240 h, the L* values of all groups converged, with no statistically significant differences among them (*p* > 0.05), indicating that the darkening effect induced by prolonged fermentation overrides initial treatment differences. In addition to these changes, both a* and b* values showed transient increases during the first 72 h of fermentation, followed by a gradual decrease toward the end of the process.

This color change may be attributed to some polyphenol oxidases which can cause oxidation processes and browning of phenolic compounds, resulting in color changes [22,23,46]. While beneficial for preservation and bioactivity, the fermentation influences the visual quality of *H. erinaceus*, which may be a factor to consider in consumer acceptance since darker appearance can be associated with spoilage. Thus, future applications of fermented *H. erinaceus* may benefit from incorporating the material into composite foods, where color is less critical to perception.

Rheological measurements showed a pronounced reduction in gel strength (G′) across all treatments, from ~17 kPa at the start to less than 3 kPa at the end of fermentation (Figure 3). The corresponding visual transition from a dense, heterogeneous structure to a homogeneous, liquid-like texture (Figure 4) reflects acid-induced destabilization of the mushroom polysaccharide-protein matrix, as reported in other acidic fermented systems [21]. No differences between inoculated and control groups were detected, suggesting that pH-driven matrix breakdown was the dominant mechanism. Although EPS production by LAB can alter texture in dairy and plant-based systems [47,48], such effects were not evident here. Nevertheless, the resulting liquid consistency may be advantageous for specific product types, such as beverages, soups, or sauces.

The most striking outcome was the enhancement of total phenolic content (TPC) and antioxidant capacity during fermentation. All the three conditions presented an average TPC of 32 mg GAE/100 g fw at 0 h. Inoculated treatments, particularly *Lp*, displayed significantly higher TPC values (48.3 mg GAE/100 g fw at 240 h) compared to the control (40.2 mg GAE/100 g fw). Parallel increases were observed in antioxidant assays, especially DPPH and ABTS, which are highly correlated (r = 0.94, *p* < 0.05). This increase in TPC coincided with a greater abundance of LAB in these samples during fermentation. These findings are consistent with previous studies reporting that the application of LAB, particularly *Lp* strains but also *Lc*, can enhance TPC [14,49].

These improvements can be attributed to microbial enzymatic activities, including β-glucosidase, which hydrolyzes glycosidic bonds and releases bound phenolic compounds [12,14,49,50]. Similar fermentation-driven enhancements of antioxidant properties have been documented in apple juice, blueberry pomace, and other plant substrates [13,14,15,49]. The consistency of our findings with this literature supports the conclusion that LAB-mediated fermentation can transform *H. erinaceus* by-products into antioxidant-rich ingredients.

The monitoring of biogenic amines (BAs) is particularly relevant in fermented foods because their presence directly impacts food safety and quality. Certain BAs, including histamine and tyramine, are well known for their toxicological effects, with excessive intake leading to headaches, hypertension, or allergic-like reactions. Other amines such as putrescine and cadaverine, although less toxic themselves, can enhance the harmful effects of histamine and tyramine by interfering with detoxifying enzymes [51]. For this reason, the accumulation of these compounds in the control fermentation highlights the risks associated with spontaneous microbial activity. Conversely, polyamines such as spermidine and spermine are naturally occurring metabolites with recognized physiological benefits. They are involved in cellular growth and autophagy, and dietary spermidine intake has been linked to protection against age-related memory decline [26] and reduced cardiovascular risk [28]. In this study, *Lp. plantarum*–fermented samples consistently exhibited higher spermidine concentrations, suggesting that controlled fermentation not only reduces the risk of toxic BA accumulation but may also enhance the nutritional and functional profile of the product. Taken together, these results underscore the importance of evaluating BA dynamics when developing fermented foods. Controlled inoculation with LAB strains represents a promising strategy to ensure safety by limiting the accumulation of harmful amines, while at the same time preserving or even increasing beneficial polyamines that contribute to the functional value of the final product.

Overall, these results reinforce the potential of *Lp. plantarum* and *Ls. rhamnosus* as effective fermentative agents in the biotransformation and valorisation of *Hericium erinaceus* by-products, highlighting the versatility of selected LAB strains as biopreservation tools. Beyond improving microbial safety and stability, fermentation enhanced the functional properties of the substrate through increased phenolic content and antioxidant activity. These improvements open possibilities for the application of fermented *H. erinaceus* by-products as functional ingredients in the formulation of health-oriented foods, such as fermented beverages, soups, sauces, or plant-based meat alternatives where both antioxidant potential and microbial stability are valued. Importantly, the valorization of mushroom by-products contributes to sustainability goals by converting perishable residues into stable, functional ingredients aligned with the circular bioeconomy [11,12,13].

Nevertheless, certain limitations should be acknowledged. Exopolysaccharide production was discussed as a potential antimicrobial and antioxidant contributor, but was not directly quantified in this study [39,40]. In addition, sensory attributes such as flavor, aroma, and color perception were not evaluated, despite their importance for consumer acceptance. Finally, pilot-scale trials are needed to confirm process robustness and to explore formulation strategies that mitigate texture loss and color changes while retaining functional benefits.

## 5. Conclusions

This study demonstrates, for the first time, that lactic acid fermentation can be applied to *Hericium erinaceus* by-products to improve both microbial stability and functional properties. Inoculation with lactic acid bacteria, particularly *Lactoplantibacillus plantarum*, ensured rapid acidification, the suppression of spoilage organisms, and enhancements of total phenolic content and antioxidant capacity. These transformations convert a highly perishable residue into a stable and bioactive ingredient, contributing to the valorization of mushroom processing waste within a circular bioeconomy framework.

A key aspect of this work was the monitoring of biogenic amines. Spontaneous fermentation led to the accumulation of potentially harmful compounds such as cadaverine and tryptamine, while inoculated fermentations maintained low levels of toxic amines and preserved or even increased beneficial polyamines like spermidine. This outcome reinforces the relevance of controlled fermentation not only for functional enrichment but also for ensuring product safety.

The findings highlight the potential of fermented *H. erinaceus* by-products for use in functional foods, nutraceutical formulations, and as natural antioxidant ingredients in clean-label products. Controlled lactic acid fermentation thus represents a sustainable and effective approach to enhance the nutritional, technological, and safety value of mushroom by-products, positioning them as innovative resources that connect food development with both health promotion and sustainability goals.

## Figures and Tables

**Table 1:** TSS (%) content and TA (g LA/100 g) during fermentation for the three different treatments.

Time (h)	Treatment	TSS (%)	TA (g LA/100 g)
0	Control	7.4 ± 0.3 ^a^	0.2 ± 0.0 ^a^
	*Lp. plantarum*	7.6 ± 0.0 ^a^	0.2 ± 0.0 ^a^
	*Ls. rhamnosus*	7.8 ± 0.1 ^a^	0.2 ± 0.1 ^a^
24	Control	7.8 ± 0.0 ^a^	0.2 ± 0.0 ^a^
	*Lp. plantarum*	8.9 ± 0.2 ^a^	0.2 ± 0.0 ^a^
	*Ls. rhamnosus*	8.1 ± 0.2 ^a^	0.2 ± 0.0 ^a^
72	Control	7.9 ± 0.0 ^a^	0.3 ± 0.0 ^ab^
	*Lp. plantarum*	8.4 ± 0.4 ^a^	0.7 ± 0.0 ^bc^
	*Ls. rhamnosus*	8.1 ± 0.0 ^a^	0.3 ± 0.0 ^ab^
144	Control	7.8 ± 0.2 ^a^	0.8 ± 0.0 ^cd^
	*Lp. plantarum*	8.8 ± 0.6 ^a^	1.0 ± 0.0 ^cde^
	*Ls. rhamnosus*	8.6 ± 0.5 ^a^	0.9 ± 0.0 ^cd^
240	Control	8.4 ± 0.0 ^a^	1.4 ± 0.2 ^cde^
	*Lp. plantarum*	8.4 ± 0.1 ^a^	1.6 ± 0.4 ^de^
	*Ls. rhamnosus*	8.4 ± 0.3 ^a^	1.7 ± 0.2 ^e^

Means followed by different letters are statistically different (*p* < 0.05).

**Table 2:** Color parameters (L*, a*, b*) and total color difference (∆E) of *Hericium erinaceus* by-products samples during 240 h of fermentation with *Lp* and *Lc*. Values are expressed as mean ± standard deviation.

Time (h)	Samples	L	a*	b*	∆E
0	Control	67.7 ± 4.5 ^bc^	1.2 ± 0.3 ^ab^	29.0 ± 3.9 ^ab^	-
	*Lp. plantarum*	72.5 ± 1.4 ^ef^	1.5 ± 0.5 ^abc^	29.8 ± 1.4 ^b^	-
	*Ls. rhamnosus*	71.7 ± 2.2 ^def^	1.5 ± 0.4 ^ab^	27.8 ± 2.6 ^ab^	-
24	Control	71.8 ± 1.0 ^def^	1.7 ± 0.2 ^abcd^	29.6 ± 3.2 ^ab^	5.0 ± 1.9 ^abc^
	*Lp. plantarum*	73.3 ± 1.3 ^f^	2.2 ± 0.6 ^cde^	33.3 ± 2.0 ^c^	4.1 ± 1.6 ^ac^
	*Ls. rhamnosus*	72.4 ± 1.1 ^ef^	2.8 ± 0.7 ^e^	34.5 ± 1.5 ^c^	7.0 ± 1.4 ^bd^
72	Control	70.5 ± 1.3 ^cdef^	3.0 ± 0.5 ^e^	33.2 ± 2.8 ^c^	6.1 ± 1.3 ^abd^
	*Lp. plantarum*	70.1 ± 0.8 ^ced^	2.4 ± 0.8 ^de^	34.1 ± 3.0 ^c^	8.3 ± 2.0 ^abc^
	*Ls. rhamnosus*	69.7 ± 1.6 ^cd^	4.3 ± 0.3 ^f^	35.2 ± 2.0 ^c^	5.2 ± 2.8 ^d^
144	Control	69.3 ± 1.6 ^bcd^	1.1 ± 0.5 ^ab^	28.6 ± 0.9 ^ab^	1.7 ± 0.5 ^c^
	*Lp. plantarum*	67.5 ± 1.2 ^bc^	1.1 ± 0.4 ^ab^	28.2 ± 0.5 ^ab^	5.0 ± 0.9 ^abc^
	*Ls. rhamnosus*	66.6 ± 1.1 ^b^	0.9 ± 0.6 ^bcd^	28.9 ± 1.1 ^ab^	5.3 ± 1.2 ^ab^
240	Control	61.7 ± 1.5 ^a^	1.1 ± 0.3 ^a^	27.5 ± 1.4 ^ab^	6.4 ± 1.6 ^abd^
	*Lp. plantarum*	61.6 ± 2.1 ^a^	1.5 ± 0.5 ^abc^	26.7 ± 1.1 ^a^	11.4 ± 2.2 ^e^
	*Ls. rhamnosus*	60.8 ± 1.6 ^a^	1.7 ± 0.3 ^abcd^	27.7 ± 1.5 ^ab^	11.0 ± 1.5 ^e^

Different superscript letters within the same column indicate statistically significant differences (*p* < 0.05).

**Table 3:** Biogenic amine content (mg/kg) in the beginning (C_i_, *Lc*_i_ and *Lp*_i_) and at the end of fermentation (C_f_, *Lc*_f_ and *Lp*_f_).

Sample	Tryptamine	Phenylethylamine	Putrescine	Cadaverine	Histamine	Tyramine	Spermidine	Spermine
C_i_	13.6 ± 2.3 ^a^	5.3 ± 3.1 ^a^	4.8 ± 2.0 ^a^	15.1 ± 0.9 ^a^	6.4 ± 0.5 ^a^	27.2 ± 0.8 ^b^	25.3 ± 0.1 ^a^	12.3 ± 0.4 ^a^
C_f_	29.7 ± 4.7 ^b^	4.0 ± 0.0 ^a^	2.3 ± 0.3 ^a^	70.3 ± 15.2 ^b^	6.6 ± 1.0 ^a^	14.8 ± 4.9 ^ab^	32.7 ± 15.9 ^a^	11.0 ± 1.1 ^a^
*Lc*_i_	10.4 ± 0.8 ^a^	0.7 ± 0.6 ^a^	2.4 ± 0.1 ^a^	7.2 ± 0.8 ^a^	5.5 ± 0.0 ^a^	9.5 ± 0.5 ^a^	29.0 ± 7.5 ^a^	11.6 ± 1.0 ^a^
*Lc*_f_	9.7 ± 0.6 ^a^	4.1 ± 0.1 ^a^	3.6 ± 0.4 ^a^	10.5 ± 0.1 ^a^	5.8 ± 0.7 ^a^	9.6 ± 2.3 ^a^	19.7 ± 10.0 ^a^	15.6 ± 3.1 ^a^
*Lp*_i_	7.4 ± 0.4 ^a^	1.4 ± 0.4 ^a^	3.0 ± 0.0 ^a^	14 ± 2.0 ^a^	6.8 ± 1.1 ^a^	12.6 ± 0.2 ^a^	45.2 ± 1.1 ^a^	10.8 ± 0.7 ^a^
*Lp*_f_	15.7 ± 0.3 ^a^	3.8 ± 1.1 ^a^	5.1 ± 1.2 ^a^	13.0 ± 0.3 ^a^	6.3 ± 0.2 ^a^	12.3 ± 0.1 ^a^	45.5 ± 11.2 ^a^	11.6 ± 1.1 ^a^

Different superscript letters within the same column indicate statistically significant differences (*p* < 0.05).

## Data Availability

The original contributions presented in this study are included in the article. Further inquiries can be directed to the corresponding author.
